# Toward Precision Cancer Immunotherapy

**DOI:** 10.1002/advs.75907

**Published:** 2026-06-15

**Authors:** Shuang Tang, Jean J. Zhao, Ada Hang‐Heng Wong

**Affiliations:** ^1^ Fudan University Shanghai Cancer Center Shanghai China; ^2^ Dana‐Farber Cancer Institute Boston Massachusetts USA; ^3^ John Wiley & Sons Inc. Maryland USA

Cancer immunotherapy has matured into a cornerstone of modern oncology. However, durable clinical benefit remains inconsistent across tumor types and patient subsets. The **Advances in Cancer Immunotherapy** Special Issue assembles reviews and original studies that collectively advance a unifying objective: transforming episodic immune activation into controllable and sustained anti‐tumor immunity through mechanistically grounded and clinically implementable strategies. Across these contributions, “precision cancer immunotherapy” emerges as the ability to achieve temporal and spatial control over immune‐evasive mechanisms while promoting the activation, trafficking, and persistence of anti‐tumor immune responses.

The articles in this Special Issue span complementary levels of biological and therapeutic control, including innate and adaptive immunity, the tumor microenvironment (TME), engineered therapy, adoptive cell therapy, and rational combination strategies. Together, they highlight how advances in mechanistic understanding, bioengineering, and translational medicine are converging to reshape the future of cancer immunotherapy.

## Harnessing Innate and Adaptive Immunity

Effective cancer immunotherapy depends not only on the magnitude of immune activation but also on the stability and reversibility of T cell states under chronic antigen exposure, as well as systemic immunity. A review reframes T cell exhaustion as a spectrum of differentiation states and regulatory programs, delineating distinct therapeutic intervention points (advs.202520634). Tumor‐primed memory T cells exhibit senescence and heightened type I interferon responsiveness that can exacerbate dysfunction during cancer vaccination, underscoring the importance of temporally coordinated priming (advs.202504474). Tissue‐resident memory CD4^+^ T cells express elevated immune checkpoint molecules and secrete the chemokine XCL‐1 to recruit dendritic cells, providing a cell‐state rationale for attenuated responses to immune checkpoint blockade in non‐small cell lung cancer (advs.202507647). Splenic remodeling during tumorigenesis is further reviewed as a determinant of systemic immunity that may influence therapeutic responsiveness (advs.202600027).

Myeloid programs also critically shape whether T cell priming and effector functions are enabled or suppressed within solid tumors. A review surveys macrophage extracellular traps as contributors to tumor progression and immune regulation (advs.202520421). In hepatocellular carcinoma, fibrates potentiate responsiveness to immune checkpoint blockade by inhibiting PLTP‐induced M2 macrophage infiltration (advs.202513257). LMO7 suppresses tumor‐associated macrophage (TAM) phagocytosis of cancer cells, revealing a molecular constraint on innate immune effector function (advs.202511162). Together, these studies highlight how immune cell state transitions and myeloid regulation govern the balance between anti‐tumor immunity and immune suppression.

## Rewriting the Tumor Microenvironment and Systemic Constraints

The TME enforces immune evasion through metabolic constraints, suppressive cellular circuits, and intercellular communication networks. Lactate‐driven signaling and protein lactylation are reviewed as mechanisms linking tumor metabolism to immune modulation and epigenetic control, while also emerging as potential therapeutic strategies in cancer immunotherapy (advs.202515403). A nanozyme targeting intratumoral *Peptostreptococcus anaerobius* reverses ferroptosis resistance, indicating that engineered modulation of local microbial niches can reconfigure tumor vulnerability (advs.202516272). Moreover, inhibition of secretion of Fgl2‐containing exosomes combined with anti‐PD‐L1 blockade prevents activation of myeloid‐derived suppressor cells (MDSCs), addressing vesicle‐mediated immune suppression as a therapeutically targetable mechanism for remodeling the TME (advs.202521784). Collectively, these studies frame metabolism, microbial context, and intercellular communication as major determinants of immunotherapy response.

## Engineered Therapies for Precision Immunotherapy

Engineered therapeutic platforms illustrate how precision immunotherapy can be achieved through designed materials, biologics, and computational frameworks that control immune exposure and programming. Manganese–DNA complex extracellular vesicles reprogram intratumoral dendritic cells to potentiate anti‐tumor immunity for pancreatic cancer (advs.202507159). An ACLY‐containing liposomal vesicle platform is used to model how engineered vesicles can induce immunosuppressive TAMs in hepatocellular carcinoma, highlighting the importance of design‐dependent immune programming (advs.202521458). Dual HLA‐G and CD47 targeting with DSP216 exemplifies disruption of immune‐evasive signaling at the tumor–immune interface (advs.202521448). Antibody–drug conjugates (ADCs) are reviewed with emphasis on how payload, linker, and target selection can be optimized for efficacy and safety (advs.202515498). Artificial intelligence (AI) and large language models (LLMs) are further discussed as enabling tools for biomarker discovery, patient stratification, and treatment optimization (advs.202521936).

## Adoptive Cell Therapy for Solid Tumors

Adoptive cell therapy enables delivery of defined immune effectors while permitting extensive *ex vivo* engineering and quality control. A review synthesizes current chimeric antigen receptor T (CAR‐T) cell therapies for solid tumors and the principal barriers that constrain their efficacy (advs.202505822). An anti‐PD‐1 nanobody–armored, mesothelin‐targeting CAR‐T cell therapy is developed for mesothelioma (advs.202508754), while humanized and charge‐optimized CSPG4‐specific CAR‐T cells demonstrate enhanced efficacy against head and neck squamous cell carcinoma (advs.202519746). A CCR4/CD7 bispecific CAR‐T modality further expands target recognition logic and antigen selection (advs.202521443). G protein‐coupled receptors (GPCRs) are reviewed as an expanded control and target space in CAR‐T cell therapy (advs.202517188). In parallel, humanization of a bispecific T cell engager is presented for central nervous system and peripheral tumors (advs.202522391).

Natural killer (NK) cell therapies are reviewed as complementary adoptive platforms (advs.75326). NK cell conjugation to IL‐15–expressing adipose‐derived mesenchymal stem cells promotes tumor localization and augments anti‐tumor efficacy (advs.202509638). Furthermore, autologous tumor‐infiltrating lymphocyte therapy (TIL) is applied in acral melanoma patients, supporting the clinical translation of patient‐specific cellular products (advs.202521555). Additionally, nanoparticle platforms for delivery of immunomodulators and chemotherapeutics are reviewed as strategies to improve adoptive cell therapies through optimized formulation and biodistribution control (advs.202522864).

## Rational Combination Regimens and Adaptive Trial Design

Because immune‐evasive mechanisms are highly redundant, durable cancer control will likely require rational combinations with deliberate dosing and sequential strategies. A review distills lessons from failed combinatorial immunotherapy trials and proposes AI‐driven, adaptive, biomarker‐guided clinical trial designs to improve learning efficiency and therapeutic decision‐making (advs.202518960). Combination of ADCs carrying anti‐tubulin and topoisomerase I inhibitor payloads with radiotherapy is evaluated as a strategy to potentiate immunotherapy and position cytotoxic modalities as programmable components of immune priming (advs.202506552). Chemo‐immunotherapy is further investigated for immune‐enriched pancreatic cancer (advs.202521844).

Vaccine‐based priming is represented by a combination of the rWTC‐MBTA vaccine with anti‐PD‐1 immunotherapy for central nervous system and peripheral B‐cell lymphoma (advs.202511605). Moreover, CDK4/6 inhibition regulates TAMs through MIF signaling to enhance CD8+ T cell anti‐tumor immunity, supporting rational combination strategies integrating immunotherapy with CDK4/6 inhibitors (advs.202511330). Pharmaceutical stimulation of β2‐adrenergic receptors further enhances cytotoxic T cell activity against *p53*‐deficient head and neck squamous cell carcinoma through CXCL10 signaling (advs.202516859). Collectively, these studies highlight rational combination strategies aimed at improving the efficacy and durability of cancer immunotherapy while informing future biomarker‐guided clinical trial design.

## Conclusion

This Special Issue underscores a clear trajectory for the field: precision cancer immunotherapy will be increasingly defined by temporally and spatially calibrated interventions that disable immune evasion while initiating and sustaining anti‐tumor immunity. Realizing this vision will require coordinated advances in mechanistic discovery, bioengineering, biomarker‐guided combination strategies, and clinical translation. Together, the studies assembled in this Special Issue illustrate how innovations across these domains are shaping the next generation of precision cancer immunotherapy.

